# Hui and Walter’s latent-class model extended to estimate diagnostic test properties from surveillance data: a latent model for latent data

**DOI:** 10.1038/srep11861

**Published:** 2015-07-07

**Authors:** Mairead L. Bermingham, Ian G. Handel, Elizabeth J. Glass, John A. Woolliams, B. Mark de Clare Bronsvoort, Stewart H. McBride, Robin A. Skuce, Adrian R. Allen, Stanley W. J. McDowell, Stephen C. Bishop

**Affiliations:** 1 The Roslin Institute and Royal (Dick) School of Veterinary Studies, University of Edinburgh, Easter Bush, Midlothian, EH25 9RG; 2 Agri-Food and Biosciences Institute Stormont, Stoney Road, Belfast, BT4 3SD, UK; 3 The Queen’s University of Belfast, Department of Veterinary Science, Stormont, Belfast BT4 3SD, UK

## Abstract

Diagnostic test sensitivity and specificity are probabilistic estimates with far reaching implications for disease control, management and genetic studies. In the absence of ‘gold standard’ tests, traditional Bayesian latent class models may be used to assess diagnostic test accuracies through the comparison of two or more tests performed on the same groups of individuals. The aim of this study was to extend such models to estimate diagnostic test parameters and true cohort-specific prevalence, using disease surveillance data. The traditional Hui-Walter latent class methodology was extended to allow for features seen in such data, including (i) unrecorded data (i.e. data for a second test available only on a subset of the sampled population) and (ii) cohort-specific sensitivities and specificities. The model was applied with and without the modelling of conditional dependence between tests. The utility of the extended model was demonstrated through application to bovine tuberculosis surveillance data from Northern and the Republic of Ireland. Simulation coupled with re-sampling techniques, demonstrated that the extended model has good predictive power to estimate the diagnostic parameters and true herd-level prevalence from surveillance data. Our methodology can aid in the interpretation of disease surveillance data, and the results can potentially refine disease control strategies.

The first line of surveillance of infectious disease is the deployment of approved and/or validated diagnostic tests which index disease occurrence and spread. A primary goal of such tests is the accurate identification of infected or non-infected individuals, which is a function of test sensitivity (the probability that the test classifies an infected individual as infected) and specificity (the probability that the test classifies an uninfected individual as uninfected^1^). The predictive value of the diagnostic test is a function of these parameters and disease prevalence.

Estimates of sensitivity and specificity are usually based on direct comparisons of test outcomes with a gold-standard which has an assumed sensitivity and specificity of 100%^2^. Unfortunately, true infection status is often impossible to determine for many reasons, including the lack of a perfect reference test. However, several statistical approaches have been developed to assess the diagnostic test accuracy in the absence of a gold standard test^3^,^4^,^5^,^6^,^7^. These methods are based on latent class probabilistic models, in which the observed status is linked to the unobserved true infection status. Estimates of sensitivities, specificities and true prevalence can then be obtained using Maximum Likelihood or Bayesian techniques^2^,^8^.

A common assumption, and one which is used in latent class analyses, is that the sensitivity and specificity of diagnostic tests are constant for each test across all subjects, and thus are independent of the circumstances of its application^9^. This assumption may not always hold as the performance of a diagnostic tests is often setting dependent. Estimates of measures of diagnostic test accuracy vary among published validation studies^10^, and this variation is often attributed to the sampling strategies used in test evaluation studies^11^. However, true differences in diagnostic test accuracy may not be directly measurable due to random and systematic errors, resulting from factors such as technical variation in test characteristics among laboratories or over time, interpretation of results, stage of disease etc. In practice, sensitivity and specificity estimates are often average values calculated from non-homogenous populations. The variation in sensitivity and specificity within and among subpopulations should therefore be addressed when applying tests at the aggregate national level^10^,^11^,^12^,^13^,^14^,^15^. This issue has been dealt with previously by assuming that the diagnostic accuracy of the imperfect reference standard is known (rather than estimating it from the data), and then performing sensitivity analysis by varying this diagnostic accuracy^15^. In this paper however, we will extend the traditional Hui-Walter latent class model^4^,^12^ to allow for the estimation of cohort specific diagnostic test properties from the data.

Disease surveillance data is a convenient source of data that may be used to evaluate or reassess diagnostic test properties, especially when combinations of tests are used in disease surveillance and eradication programmes. Such data are both convenient and relevant for assessing diagnostic test properties; however, they introduce additional complexities such as incomplete and/or missing data. The problem of missing data and verification bias of test results have been previously discussed and dealt with in the literature^14^,^16^,^17^,^18^. The ‘gold’ standard model has been extended to deal with the problem of missing data in the estimation of diagnostic test accuracy^17^. Furthermore, the subject-specific latent class method has been extended to account for verification bias in positive test results of the first test by the second test^16^. Nevertheless, missing data cannot currently be incorporated into traditional latent class models^4^,^12^. In our paper we have extended the traditional Hui-Walter model^4^,^12^ which estimates diagnostic sensitivity and specificity in the absence of a gold standard test, to include two additional multinomial counts; the probabilities that individuals deemed positive and negative by the initial diagnostic test are not classified by subsequent diagnoses.

This study extends the traditional Bayesian Hui-Walter latent class model^4^,^12^ to deal with data from surveillance studies. Specifically, we aim (i) to estimate diagnostic test parameters and true prevalence from surveillance data with some unrecorded class variables (i.e. results from a second test available only on a subset of the sampled population), and (ii) to allow for variation among sub-populations in the diagnostic test properties.

## Methods

### Specification of extended Hui-Walter Latent Class model

Here we describe two extensions to the latent class model^4^.

#### 1. Variable diagnostic test properties across cohorts

An assumption of the standard model is that the property of each test is constant across sampled cohorts. However, the performance of the tests may be modified by factors such as sources of exposure or infection pressure, different practitioners or pathogen strain(s), and cohort-specific characteristics which may vary throughout outbreaks^1^. This may be addressed by allowing test sensitivities (*S*_*e*_) and specificities (*S*_*p*_) to differ between cohorts, defining them as a population mean plus a cohort-specific deviation. Thus, for the *t*^th^ test and the i^th^ herd outbreak:


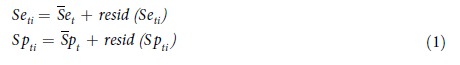


#### 2. Unrecorded data from one of the diagnostic tests

Standard latent class analyses are performed on datasets comprising individuals on which both diagnostic tests are applied. However, with surveillance data many individuals will have some unrecorded data. For example, sequential tests may be conducted on only a subset of individuals based on a positive result in the initial test, thus many negative individuals have unrecorded data for the second test. Furthermore, for numerous reasons, a subset of individuals with a positive result in the initial test will not undergo subsequent testing. Therefore, there are potentially two subsets of individuals, i.e. those with either negative or positive results from the first diagnostic test, that are not subsequently tested in the disease surveillance dataset. Hence the model may be extended to include two additional multinomial counts; the probabilities that individuals deemed positive (*T*_*2*_*n*^*+*^), and negative (*T*_*2*_*n*^*–*^) by the initial diagnostic test are not classified by subsequent diagnoses.

A further risk with diagnostic data from two (or more) tests is that the outcomes of the two tests are not independent, i.e. test results are conditional on the true disease state^12^. This concept, in which the two tests are not independent assessments of the underlying infection status, is known as conditional dependence. In this case, the covariance structure between the two tests^2^,^12^,^19^ may be included in the model, in addition to herd-level variability in diagnostic properties. For example, the probability that two tests (*T*_*1*_ and *T*_*1*_) are both positive can be written as:


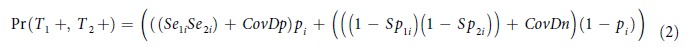


Where *p*_*i*_ is the true prevalence in the *i*^th^ herd outbreak, and *covDp* and the *CovDn* are the covariances between outcomes of the two diagnostics conditional upon infection status, when the individual is infected, and when it is not infected, respectively.

The probabilities of observing each of the six diagnostic combinations in the *i*^th^ herd outbreak can be written (with all terms previously defined) as:


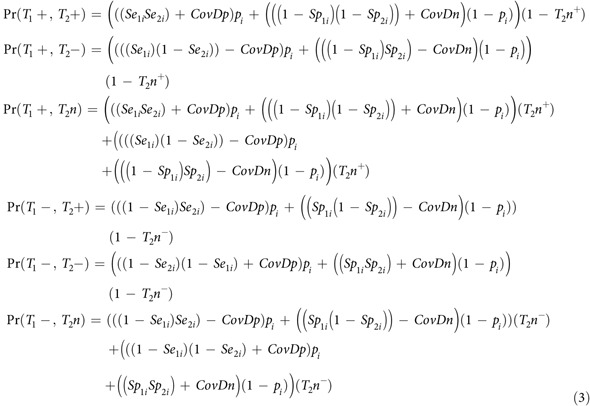


The procedure to obtain numerical solutions to equation set (3) is given in suplementary materials (text S1).

### Application of extended model to disease surveillance data

In Northern Ireland (NI) bovine tuberculosis surveillance involves annual tuberculin skin testing of all cattle, using the single intradermal comparative tuberculin test (SICTT), plus additional risk-based testing and compulsory slaughter of test reactor cattle, followed by post-mortem abattoir inspection of all animals for tuberculosis lesions. All SICTT and abattoir surveillance data from this program are recorded by the NI Department of Agriculture and Rural Development (DARD)^20^. The four cell^8^ and extended six cell models were applied to these surveillance data, with the two diagnostic tests being SICTT and subsequent abattoir inspection records on a subset of animals with SICTT measurements. The constructed dataset contained fewer abattoir inspection records than SICTT measurements for two reasons. Firstly, only abattoir inspection records obtained within 45 days of a positive SICTT result were included in the dataset^21^. Positive post-mortem records outside this window were ignored, as it is possible that animals became infected after the last SICTT. Further, a small subset of positive SICTT animals may not have an abattoir record if they had not been subject to meat inspection, such as animals that had been sent for rendering (e.g. animals over the age limit for human consumption). Individual herd outbreak size in this study was chosen to give an absolute precision for parameter estimation of 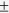
5% (see text S2) and herd outbreaks not meeting this criterion were excluded. A sample of 7920 SICTT records (614 positive results) from Holstein-Friesian dairy cows, of which 3090 had valid abattoir inspection records (215 positive results) within the specified period, from 41 bovine tuberculosis outbreaks of the required size, were extracted from DARD’s animal health database, anonymised and used for analysis.

The assumption of conditional independence may be violated if, e.g., outcomes of the first test are used as part of the procedure for interpreting the second test results, or if the two tests measure the same aspect of the host response, thereby allowing both tests to be biased by the same external factor. The SICTT and abattoir inspection are based on different principles: detection of cellular immune response vs. visual gross pathological evidence of *Mycobacterium bovis* infection, thereby reducing the risk conditional dependence between tests. To assess the ability of the extended model to estimate diagnostic test properties in the case where there is a higher risk of conditional dependence, SICTT and interferon(IFN)-γ assay data (both based on detection of cellular immune response^12^) from Republic of Ireland (RI) data were used. A sample of 2,089 SICTT records (241 positive results) from cattle, of which all had valid IFN-γ test records (474 positive results) within the specified period from 38 RI tuberculosis outbreaks (of required sample size) were selected and used for model validation. There were no missing records in this dataset.

The data for each outbreak comprised 6 multinomial counts, with T_1_^+^ T_2_^+^, T_1_^+^ T_2_^–^, T_1_^–^ T_2_^+^ and T_1_^–^ T_2_^–^ denoting the numbers for each of the 4 cross-classification levels of SICTT and abattoir inspection (IFN-γ test), and T_1_^+^ T_2_n and T_1_^–^ T_2_n, denoting the number of SICTT positive and negative cows with no abattoir inspection report within the specified period, respectively (Table 1,2).

### Testing for biases and sampling properties in estimation

An empirical test of the impact of prevalence on parameter estimates was performed by sampling further herds and stratifying the data into low, (≤0.10), moderate (0.20–0.30) and high (≥0.40) estimated true prevalence herd outbreaks; results were subsequently compared in the 3 prevalence strata. Bayesian jackknife and bootstrap analyses were used to estimate bias, and sampling distributions, respectively, of parameter estimates from the extended six cell model across the original 41 sampled herd outbreaks (see text S3).

A simulation study was conducted to test the predictive ability of the model. Data for the 6 multinomial counts, with T_1_^+^ T_2_^+^, T_1_^+^ T_2_^−^, T_1_^−^ T_2_^+^, T_1_^−^ T_2_^−^, T_1_^+^ T_2_n and T_1_^−^ T_2_n, were simulated (i.e. reconstructed) for each herd. The latent class parameter estimates from this study and Clegg *et al.*^22^ were substituted into the extended probability equations of observing each of the six diagnostic combinations, at true prevalence levels of 0.05, 0.10, 0.20, 0.40 and 0.80. The reconstructed data were then analysed using the four and extended six cell conditional independence and dependence models, as described above. The predictive power of the model was measured by correlating the herd-specific input parameter values with those estimated from the simulated data.

## Results

### Application of the extended latent class model

Models with herd-specific specificity were not considered further, as the realised specificity was close to unity, making the between-herd variability insignificant.

Results from different formulations of the Hui-Water model for the SICTT and abattoir inspection from the NI dataset are given in table 3 and 4. The results were obtained from a subset of the available data and should not be considered definitive parameter estimates. The inclusion of conditional dependence between the diagnostic test results reduced the precision of the estimates and failed to improve the fit of the model to the data (DIC <2^23^; ), with the 95% credibility intervals for the covariance parameters from both models including zero^12^, indicating that conditional independence is a valid assumption. When the sensitivities of SICTT and abattoir inspection were modelled for each outbreak in the conditional independence model the cohort specific parameters varied (Supplementary Figure S1-2), and the model fit was markedly better than when sensitivities were fixed across outbreaks, with the DIC of these models decreasing by 94.2 and 101.3 for the four, and six cell models respectively. For reference, differences of 10 or greater lead to unambiguous exclusion of the model with the higher DIC^24^. Further, when outbreak-specific SICTT and IFN-γ assay sensitivities were modelled in the RI data, cohort specific model parameters varied (Supplementary Figure S3), and the model fit was markedly better than when sensitivities were fixed across outbreaks (a reduction in DIC of 48.1; Table 5).

### Parameter estimates from the Northern Ireland bTB surveillance data

**Figure 1:**
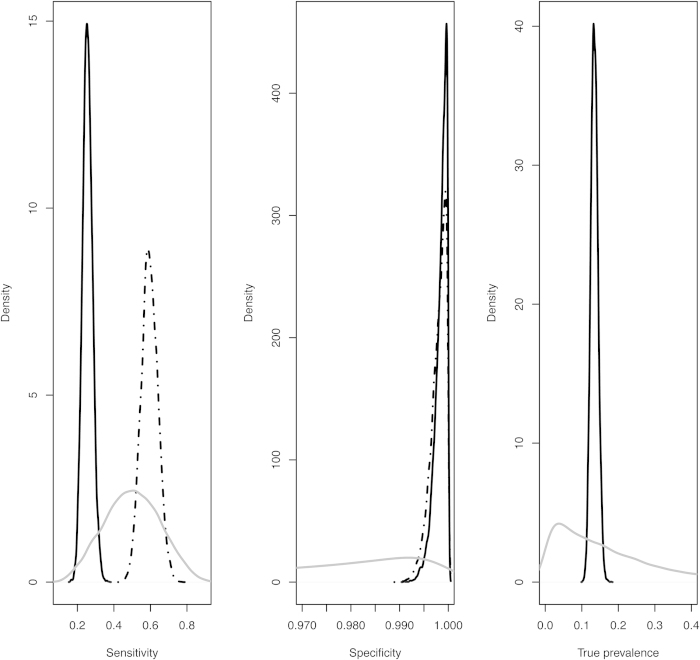
Estimated posterior distributions of parameters from the conditional independence model with outbreak specific diagnostic sensitivities from the Northern Ireland bovine tuberculosis surveillance data. The plots depict the posterior distributions of diagnostic sensitivity and specificity of the single intradermal comparative tuberculin test (broken black line) and abattoir inspection (black line), and average true prevalence from the three Markov chain Monte Carlo chains run. The grey lines represent the prior distributions used to inform the estimates. The x-axis provides the parameter estimates and the y-axis the relative probability of taking a given value.

The estimated full posterior probability distributions of the test parameters are given in Fig. 1. The distributions also demonstrate that posterior estimates differ from the prior distributions of the parameter estimates; i.e. the posterior estimates were driven by the data and not dominated by the assumed prior distributions.

Estimated true prevalence levels did not markedly affect the estimated parameter values. The sensitivity and specificity estimates from herd outbreaks stratified by estimated true prevalence were similar and the 95% credibility intervals showed substantial overlap between the three data subsets (Table 6).

Jackknife analysis demonstrated that parameter estimates from the extended model were not biased by any particular herd outbreak; 98.5%, and 100% of the jackknife estimates (n = 205, comprising 41sensitivity and specificity estimates for both tests and average true prevalence) were within 1 and 2 standard deviations of the full dataset estimates. The estimates for sensitivity, specificity were also robust to removal of 25% population subsets; the 95% bootstrap confidence interval of the empirical distribution was marginally tighter than that of the diagnostic parameter estimates (Supplementary Table S1).

### Simulation study

**Figure 2:**
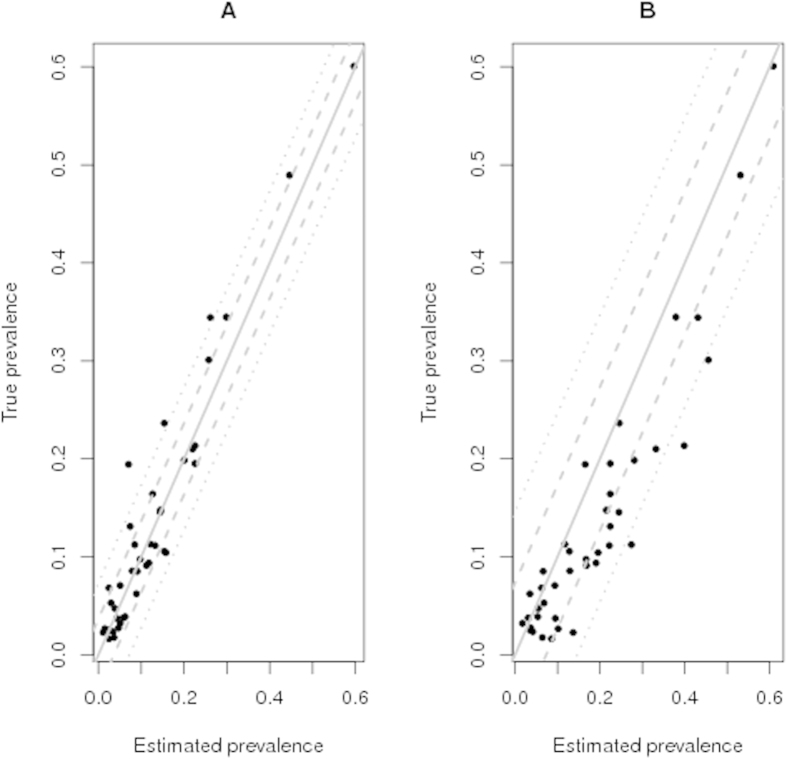
The relationship between input and output estimated true prevalence values from the extended six (**A**) and traditional four (**B**) cell conditional independence including outbreak specific sensitivities from the simulation study. The grey line superimposed onto the plot represents a regression coefficient of 1 between the parameters (with the dashed and dotted grey lines representing one and two root mean square errors from the regression line respectively).

**Figure 3:**
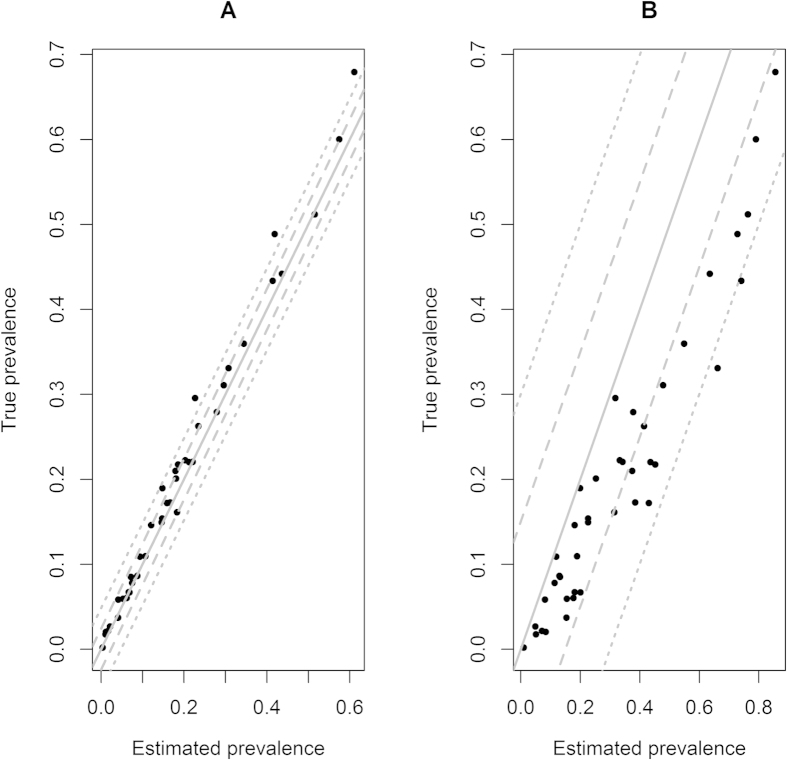
The relationship between input and output estimated true prevalence values from the extended six (**A**) and traditional four (**B**) cell conditional dependence models including outbreak specific sensitivities from the simulation study. The grey line superimposed onto the plot represents a regression coefficient of 1 between the parameters (with the dashed and dotted grey lines representing one and two root mean square errors from the regression line respectively).

**Figure 4:**
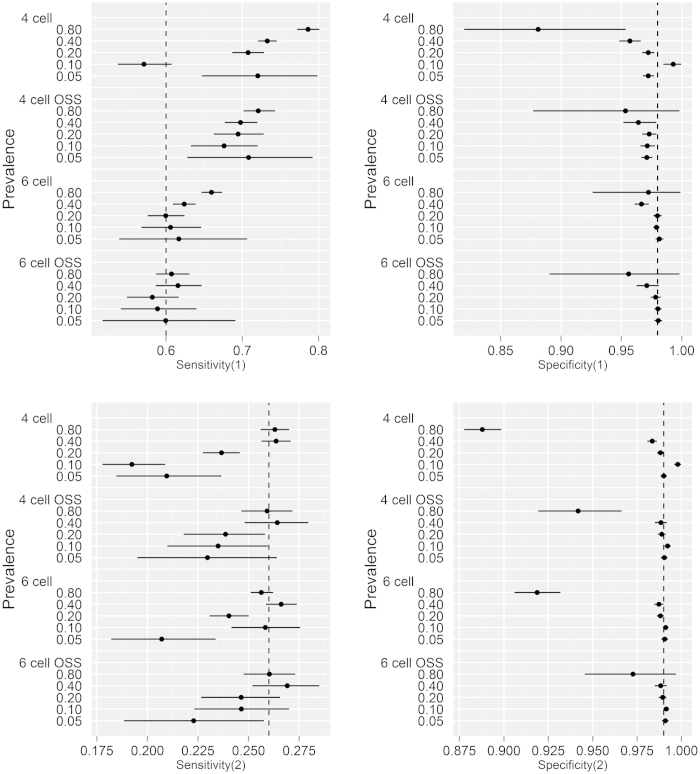
Parameter estimates (with 95% Bayesian credibility intervals) of diagnostic sensitivity and specificity for the single intradermal comparative tuberculin test (**1**) and abattoir inspection (**2**) from the simulated data sets from the traditional 4 and extended 6 cell conditional independence model excluding/including outbreak specific sensitivities (OSS). The simulated data was reconstructed using parameter estimates from the Northern Ireland surveillance data; with true prevalence ranging from 0.05 to 0.80. The broken perpendicular lines on each of the plots represent the simulation input value for the respective parameters. Modelling OSS improved the precision; whereas the 6 cell extension augmented the accuracy of the parameter estimates in the absence of conditional dependence between the diagnostic tests.

**Figure 5:**
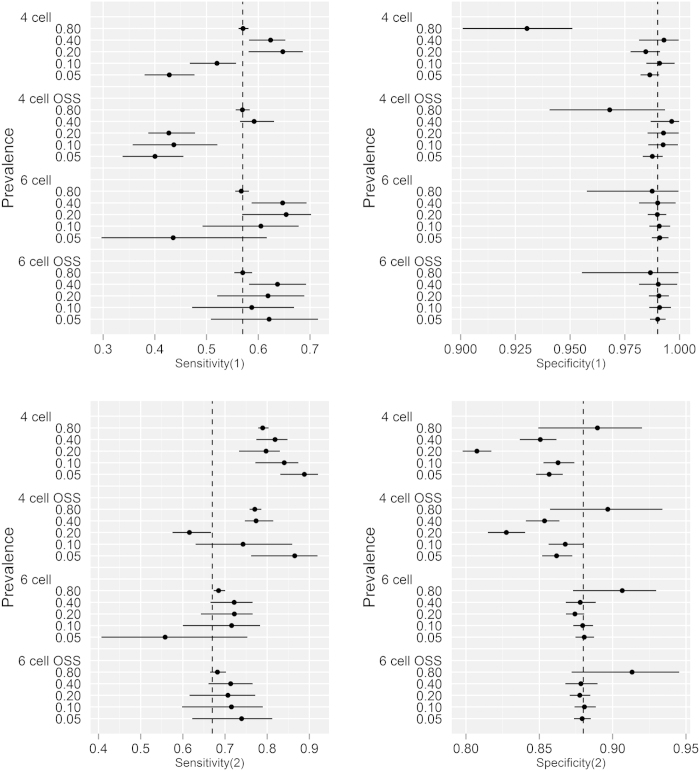
Parameter estimates (with 95% Bayesian credibility intervals) of diagnostic sensitivity and specificity for the single intradermal comparative tuberculin test (**1**) and abattoir inspection (**2**) from the simulated data sets from the traditional 4 and extended 6 cell conditional dependence model excluding/including outbreak specific sensitivities (OSS). The simulated data was reconstructed using parameter estimates from Clegg *et al.*^22^; with true prevalence ranging from 0.05 to 0.80. The broken perpendicular lines on each of the plots represent the simulation input value for the respective parameters. Modelling OSS improved the precision; whereas, the 6 cell extension augmented the accuracy of the parameter estimates in the absence of conditional dependence between the diagnostic tests.

**Figure 6:**
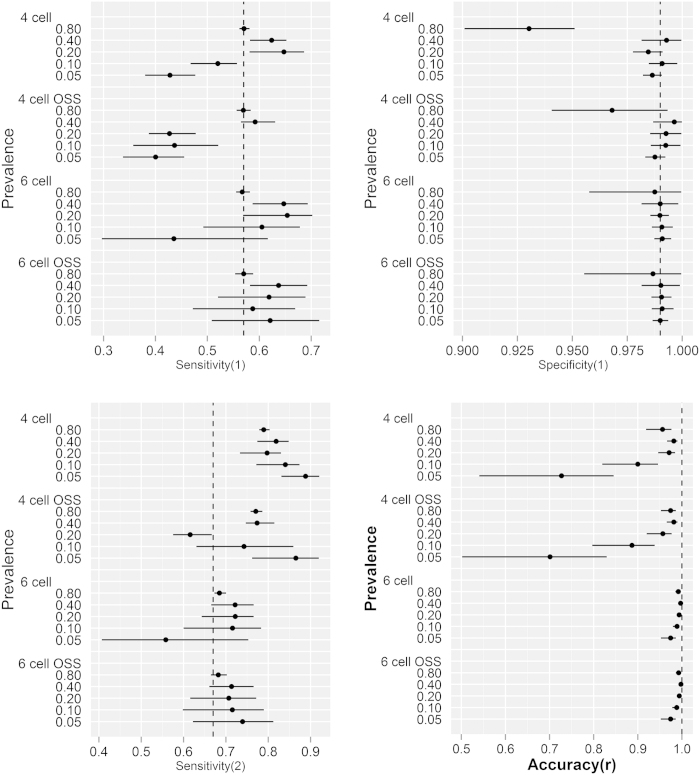
Parameter estimates (with 95% Bayesian credibility intervals) of prevalence and accuracy (r) of predicting within cohort prevalence from the simulated data sets from the traditional 4 and extended 6 cell conditional independence and dependence models excluding/including outbreak specific sensitivities (OSS). The simulated data for the conditional independence and dependence models were reconstructed using parameter estimates from the Northern Ireland surveillance data and Clegg *et al.*^22^ respectively; with true prevalence ranging from 0.05 to 0.80. The broken perpendicular lines on the upper plots represent the simulation input value for true prevalence, and those on the lower plots depict an accuracy of 1. Modelling OSS had little impact on the precision of dataset and within cohort prevalence estimates. The 6 cell extension improved the accuracy of the estimates of dataset and within cohort prevalence in the absence/presence of conditional dependence between the diagnostic tests.

The simulation study demonstrated that the extended six cell conditional latent class model, with and without conditional dependence between the tests, has good predictive power to estimate diagnostic parameters of the SICTT, abattoir inspection and IFN-γ assay from these surveillance data and for the parameter ranges explored. There was no significant bias observed in the estimated sensitivity and specificity of the tests and the 95% credibility intervals of the simulated diagnostic parameters overlapped widely with the posterior distribution of the diagnostic parameter estimates (Table 7,8). In the simulation study, the four cell conditional independence model generated posterior distributions of SICTT sensitivity and true prevalence that were very similar to those obtained from the surveillance data (Table 3); indicating these parameters were biased upwards, in contrast to those from the six cell model. The posterior distribution of abattoir inspection sensitivity was unbiased (Table 7). The simulation study also demonstrated that the extended six cell conditional independence model had strong predictive power to estimate true herd prevalence. The correlation between the input and output estimated herd-specific prevalence was 0.96 (95% CI 0.93–0.98) and 0.99 (95% CI 0.90–1.00) from the conditional independence and dependence models correspondingly. The four cell latent class model gave marginally lower correlations between the input and output estimated prevalence, viz. 0.93 (95% CI 0.88–0.96) and 0.97(95% CI 0.95–0.98) from the conditional independence and dependence models, respectively. The four cell model was less precise with a root mean square prediction error (RMSE) for herd-specific prevalence estimates of 0.073 and 0.150, compared to 0.036 and 0.024 from the six cell conditional independence and dependence models. Furthermore, the six cell conditional independence (dependence) model had greater accuracy compared to the four cell models, 27% (24%) as opposed to 34% (48%) of the prevalence estimates were one RMSE from the regression line, respectively (Figs 2 and 3). The true prevalence levels in the simulated data had marked effects on the estimated parameter values across the different models (detailed results in Figs 4, 5, 6). Modelling individual out-break sensitivities improved the precision of the diagnostic test parameter estimates to some extent. However it was the six cell extension that removed the dependence of the modelled parameters on the prevalence.

## Discussion

Surveillance data are collected routinely by national authorities; validated diagnostic tests are deployed for the purpose of detecting incursions of, demonstrating freedom from, or assessing changes in disease incidence^25^. These data provide a valuable resource with which to assess the performance of diagnostic tests within population-level surveillance programmes. However, to date, assessment of diagnostic test performance has been constrained to sample-based evaluation, as the traditional Hui-Walter latent class models^4^,^12^ applied can only handle simple data structures, such as the data collected in traditional diagnostic evaluation studies. In this study we extended the traditional Hui-Walter latent class model, allowing for missing data and between-herd variability in diagnostic properties, potentially exploiting available surveillance data resources, to generate population-based estimates at the regional or national level. For demonstration purposes we have only used a subset of the available data; a larger dataset may have enabled us to fit more complex models with greater robustness.

Bayesian and frequentist maximum-likelihood approaches have been used to fit the traditional Hui-Walter latent class model^3^,^4^,^22^,^26^,^27^. It was beyond the scope of the project to extend the model within two frameworks. We therefore chose to use a Bayesian approach to extend the traditional Hui-Walter latent class model; because of the expertise of our project members^2^,^8^. Moreover, Bayesian and maximum-likelihood approaches would have produced equivalent results in this study; because of the large number of subpopulations with different prevalences included in the analyses^26^,^27^.

Fitting the extended six cell Hui-Walter latent class model to bovine tuberculosis surveillance data, we found the extended model to give robust results, particularly when between-herd variability in sensitivity was modelled. Through simulation-based analyses, the study also demonstrated that the extended six cell model outperformed the four cell latent class model in in terms of improved predictive ability and reduced bias of estimated diagnostic parameters. In practice, the diagnostic accuracy of a test may be dependent on the disease prevalence in the population tested^13^,^28^,^29^. However, this was not observed here, as the prevalence strata-specific estimates of diagnostic accuracy did not differ in this study.

Diagnostic accuracy is frequently assumed to be constant across populations. However this is seldom the case, as results may be influenced by factors such as whether the disease is clinical or subclinical, pathological progression, time since infection, immune status, prevalence of cross-reacting organisms and operator error^1^,^11^,^30^,^31^. Furthermore, the tests are undertaken by many practitioners, under variable conditions, and in such circumstances it is difficult to avoid inconsistencies in performance^31^. These factors may vary within populations, hence there may be substantial variation heterogeneity in sensitivity (or specificity) estimates depending on the population and animal level sampling plan^11^. A novel aspect of our analysis was that diagnostic sensitivity was allowed to vary across herd outbreaks within the traditional Hui-Walter latent class model framework^4^,^12^. This extension proved useful in improving the goodness of fit of the model, capturing variation in diagnostic sensitivity between outbreaks. Specificities of the two diagnostic tests may also vary across cohorts, as specificity is particularly susceptible to the characteristics of the population and settings including, for example, geographical variation in cross reacting organisms^1^,^10^,^32^. However, it was not possible to model variable specificities in this dataset, as the estimated parameters were close to unity.

It is generally recommended that diagnostic test accuracy be validated carefully before being applied to field surveillance data, particularly if disease control programmes are subsequently based on these data. Here we present novel methodologies which may allow this to be done, and also which maximise the utility of existing data.

## Conclusion

This study has provided novel extensions to the traditional Hui-Walter latent class model that can aid in the interpretation of disease surveillance data. The extended methodology developed in this study can be used to continually monitor diagnostic test performance, and to identify systematic factors which may reduce their efficacy through strata-specific analyses using readily available surveillance data. This extended methodology also has applications in the epidemiological analysis of diseases for which incomplete surveillance data on two or more diagnostic tests are available.

### Ethics statement

The methods were carried out in accordance with the approved guidelines. All experimental protocols were approved by the Department of Health, Social Security and Public Safety for Northern Ireland (DHSSPSNI) under the UK Animals (Scientific Procedures) Act 1986 [ASPA], following a full Ethical Review Process by the Agri-Food & Biosciences Institute (AFBI) Veterinary Sciences Division (VSD) Ethical Review Committee. The study is covered by DHSSPSNI ASPA Project Licence (PPL-2638 ‘Host Genetic Factors in the Increasing Incidence of Bovine Tuberculosis’), and scientists and support staff working with live animals during the studies all hold DHSSPSNI ASPA Personal Licences.

## Additional Information

**How to cite this article**: Bermingham, M. L. *et al.* Hui and Walter’s latent-class model extended to estimate diagnostic test properties from surveillance data: a latent model for latent data. *Sci. Rep.* **5**, 11861; doi: 10.1038/srep11861 (2015).

## Supplementary Material

Supplementary Information

## Figures and Tables

**Table 1:** Tabulation of total concordant, discordant single intradermal comparative tuberculin test (SICTT) and abattoir inspection, and SICTT with non-observed abattoir inspection diagnostic results across all 41 Northern Ireland tuberculosis herd outbreaks.

		Test 2: Abattoir inspection		
		Positive	Negative	Unrecorded
Test 1: SICTT	Positive	173	408	33
	Negative	42	2,467	4,797

**Table 2:** Tabulation of total concordant, discordant single intradermal comparative tuberculin test (SICTT) and interferon(IFN)-γ assay across all 38 Republic of Ireland tuberculosis herd outbreaks.

		Test 2: Interferon(IFN)-γ assay	
		Positive	Negative
Test 1: SICTT	Positive	193	48
	Negative	281	1,567

**Table 3:** Results from four cell models: parameter estimates (with 95% Bayesian credibility intervals) of diagnostic sensitivity (Se) and specificity (Sp) for the single intradermal comparative tuberculin test (1) and abattoir inspection (2), negative (CovDn) and positive (CovDp) covariances between the two test, average true prevalence (P) and deviance information criterion (DIC) from the traditional four cell conditional independence and dependence models excluding/including outbreak specific sensitivities (–/+ OSS).

Model	Without OSS		With OSS
Parameter	Independence	Dependence	Independence
Se_1_	0.814_(0.759–0.869)_	0.820_(0.711–0.915)_	0.767_(0.703–0.833)_
Sp_1_	0.979_(0.962–0.995)_	0.971_(0.952–0.9887)_	0.988_(0.973–0.999)_
Se_2_	0.329_(0.285–0.376)_	0.329_(0.270–0.392)_	0.269_(0.215–0.331)_
Sp_2_	0.998_(0.993–1.000)_	0.994_(0.985–0.999)_	0.999_(0.995–1.000)_
CovDn	–	0.004_(0.000–0.011)_	–
CovDp	–	_–_0.005_(–0.044–0.031)_	–
P	0.178_(0.157–0.201)_	0.170_(0.144–0.200)_	0.195_(0.172–0.217)_
DIC	559.36	557.69	465.22

**Table 4:** Results from six cell models: parameter estimates (with 95% Bayesian credibility intervals) of diagnostic sensitivity (Se) and specificity (Sp) for the single intradermal comparative tuberculin test (1) and abattoir inspection (2), negative (CovDn) and positive (CovDp) covariances between the two test, average true prevalence (P) and deviance information criterion (DIC) from the six cell conditional independence and dependence models excluding/including outbreak specific sensitivities (_–_/+ OSS).

Model	Without OSS		With OSS
Parameter	Independence	Dependence	Independence
Se_1_	0.645_(0.568–0.726)_	0.604_(0.491–0.748)_	0.595_(0.508–0.687)_
Sp_1_	0.996_(0.992–1.000)_	0.995_(0.988–1.000)_	0.998_(0.994–1.000_
Se_2_	0.307_(0.268–0.350)_	0.281_(0.218–0.359)_	0.256_(0.205–0.310)_
Sp_2_	0.998_(0.993–1.000)_	0.996_(0.991–1.000)_	0.999_(0.995–1.000)_
CovDn	_–_	0.001_(0.000–0.003)_	_–_
CovDp	_–_	0.014_(−0.036–0.045)_	_–_
P	0.122_(0.106–0.139)_	0.129_(0.103–0.157)_	0.135_(0.117–0.155)_
DIC	1019.11	1018.57	917.80

**Table 5:** Parameter estimates (with 95% Bayesian credibility intervals) of diagnostic sensitivity (Se) and specificity (Sp) for the single intradermal comparative tuberculin test (1) and interferon(IFN)_–_γ assay (2), negative (CovDn) and positive (CovDp) covariance between the two test, average true prevalence (P) and deviance information criterion (DIC) from the traditional four cell conditional independence and dependence models excluding/including outbreak specific sensitivities (_–_/+ OSS).

Parameter	Model	
	Without OSS	With OSS
Se_1_	0.525_(0.451,0.600)_	0.554_(0.482,0.626)_
Sp_1_	0.998_(0.993,1.000)_	0.998_(0.993,1.000)_
Se_2_	0.813_(0.728,0.881)_	0.872_(0.810,0.918)_
Sp_2_	0.938_(0.918,0.958)_	0.937_(0.917,0.956)_
CovDn	0.001_(0.000,0.003)_	0.001_(0.000,0.003)_
CovDp	_−_0.008_(–0.046,0.035)_	_−_0.029_(–0.053,0.006)_
P	0.193_(0.171–0.219)_	0.167_(0.151,0.186)_
DIC	466.62	418.471

**Table 6:** Parameter estimates (with 95% Bayesian credibility intervals) of diagnostic sensitivity (Se) and specificity (Sp) for the single intradermal comparative tuberculin test (1) and abattoir inspection (2) from the six cell conditional independence and dependence models including outbreak specific sensitivities after the surveillance data was stratified in to low (n = 41), median (n = 41) and high (n = 17) true prevalence (P) subsets.

Parameter				
P	Se_1(BCI)_	Sp_1(BCI)_	Se_2(BCI)_	Sp_2(BCI)_
Low	0.498_(0.406–0.597)_	0.998_(0.994–0999)_	0.249_(0.184–0.312)_	0.999_(0.997–1.000))_
Median	0.617_(0.559–0.676)_	0.999_(0.995–1.000)_	0.255_(0.219–0.293)_	0.999_(0.996–1.000)_
High	0.527_(0.472–0.583)_	0.996_(0.984–0.999)_	0.245_(0.215–0.278)_	0.997_(0.990–0.999)_

**Table 7:** Parameter estimates of diagnostic accuracy (with 95% Bayesian credibility intervals) for the single intradermal comparative tuberculin test (1) and abattoir inspection (2) from the simulated data set (reconstructed using parameter estimates from the surveillance data) from the traditional 4 and extended 6 cell conditional independence model including outbreak specific sensitivities.

Conditional independence model			
	Input	Model estimates	
Parameter	6 cell	4 cell	6 cell
Se_1_	0.60	0.741_(0.667–0.825)_	0.596_(0.508–0.692)_
Sp_1_	0.98	0.994_(0.957–0.992)_	0.994_(0.988–0.999)_
Se_2_	0.26	0.249_(0.202–0.230)_	0.287_(0.233–0.347)_
Sp_2_	0.99	0.997_(0.993–1.000)_	0.998_(0.993–1.000)_
P	0.10	0.186_(0.162–0.212)_	0.128_(0.109–0.148)_

**Table 8:** Parameter estimates of diagnostic accuracy (with 95% Bayesian credibility intervals) for the SICTT (1) and abattoir inspection (2) from the simulated data set (reconstructed using parameter estimates from Clegg*et al.*^22^) from the traditional 4 and extended 6 cell conditional independence model including outbreak specific sensitivities.

Conditional dependence model			
	Input^22^	Model estimates	
Parameter	6 cell	4 cell	6 cell
Se_1_	0.5700	0.648_(0.581–0.686)_	0.619_(0.520–0.689)_
Sp_1_	0.9900	0.985_(0.997–0.991)_	0.991_(0.986–0.995)_
Se_2_	0.6700	0.797_(0.733–0.830)_	0.707_(0.616–0772)_
Sp_2_	0.8800	0.810_(0.798–0.817)_	0.878_(0.871–0.885)_
CovDn	0.0039	0.063_(0.047–0.074)_	_−_0.026_(–0.069–0.028)_
CovDp	0.0071	0.006_(0.000–0.011)_	0.007_(0.004–0.011)_
P	0.2000	0.326_(0.289–0.360)_	0.189_(0.168–0.223)_
