# Supplementary material for: Hui and Walter’s latent-class model extended to estimate diagnostic test properties from surveillance data: a latent model for latent data
Source: Sci Rep. 2015 Jul 7;5:11861. doi: 10.1038/srep11861 (PMC4493568; doi:10.1038/srep11861)
Supplement: Supplementary Information [file srep11861-s1.doc]

# Hui and Walter's latent-class model extended to estimate diagnostic test properties from surveillance data: a latent model for latent data

(Revised for Scientific Reports)

Mairead L., Bermingham^1*^, Ian G. Handel^1^, Elizabeth J. Glass^1^, John A. Woolliams^1^, B. Mark de Clare Bronsvoort^1^, Stewart H. McBride^2^, Robin A. Skuce^2,3^, Adrian R. Allen^2^, Stanley W. J. McDowell^2^, and Stephen C. Bishop^1^

^1^The Roslin Institute and Royal (Dick) School of Veterinary Studies, University of Edinburgh, Easter Bush, Midlothian, EH25 9RG.

^2^Agri-Food and Biosciences Institute Stormont, Stoney Road, Belfast, BT4 3SD, U.K.

^3^The Queen’s University of Belfast, Department of Veterinary Science, Stormont, Belfast BT4 3SD, U.K.

* To whom correspondence should be addressed.

Mairead Bermingham,

MRC Human Genetics Unit,

MRC IGMM,

University of Edinburgh,

Western General Hospital,

Crewe Road,

Edinburgh,

EH4 2XU,

UK.

Email: mairead.bermingham@igmm.ed.ac.uk

Phone: +44 131 3322471

**Supplementary Methods:**

**Text S1.** **Obtaining numerical solutions to the Latent Class analysis**

A Bayesian approach was adopted to solve the equations; model parameters were estimated by numerically integrating over the joint posterior distribution of estimated true prevalence in each outbreak and parameters of the SICTT and abattoir inspection diagnosis. Estimates from the literature were used to generate mildly informed beta priors for the diagnostic parameters. The sensitivities of the diagnostic tests were given vaguely informed beta (5, 5) priors with a mean of 0.503 and a range of 0.104 to 0.884. Each test specificity was given a mildly informed beta (50, 1) prior with a mean of 0.981 and range of 0.887 to 1.000.

The covariance between the two test outcomes for infected subpopulations satisfies $\left(Se_{1}-1\right)\left(1-Se_{2}\right)\leq\mathrm{cov}Dp\leq\left(\min\left[Se_{1},Se_{2}\right]-\left(Se_{1}Se_{2}\right)\right)$ and for non-infected subpopulations, $\left(Sp_{1}-1\right)\left(1-Sp_{2}\right)\leq\mathrm{cov}Dp\leq\left(\min\left[Sp_{1},Sp_{2}\right]-\left(Sp_{1}Sp_{2}\right)\right)$[^3^](#_ENREF_3). Therefore, uniform $\left(\left(Se_{1}-1\right)\left(1-Se_{2}\right),\left(\min\left[Se_{1},Se_{2}\right]-\left(Se_{1}Se_{2}\right)\right)\right)$ and uniform $\left(\left(Sp_{1}-1\right)\left(1-Sp_{2}\right),\left(\min\left[Sp_{1},Sp_{2}\right]-\left(Sp_{1}Sp_{2}\right)\right)\right)$ prior distributions can be used for *CovDp* and *CovDn*, respectively [^4^](#_ENREF_4).

The prior distributions for the outbreak-specific prevalence and residuals for the diagnostic test sensitivities were given vague beta (1, 5) and uniform (-0.20, 0.20) priors respectively, as there were no available data to inform these estimates. The model was implemented in WinBUGS software [^5^](#_ENREF_5) using the R2Winbugs package [^6^](#_ENREF_6), run in the R environment. WinBUGS uses a MCMC sampling algorithm to obtain the posterior distribution.

Three MCMC chains were run for the analysis to provide MCMC diagnostics. The first 500,000 iterations were discarded as burn-in to allow convergence. The subsequent 500,000 iterations were retained and thinned to 10,000 for posterior inference. The convergence of the chains following the initial burn-in period was assessed by visual inspection of the time series plots for the parameter samples, and the Gelman-Rubin diagnostic plots using three sample chains with different starting values [^7^](#_ENREF_7). Posterior inference was done by calculating means and 95% credibility intervals, of the prevalence across herd outbreaks, and the diagnostic parameters of the SICTT and abattoir inspection. Analysis and graphing of the MCMC output was conducted in the R package CODA [^8^](#_ENREF_8).

The models with and without conditional independence between the diagnostic results of the SICTT and abattoir inspection were compared using the Deviance Information Criteria (DIC), which is a composite measure:$\text{DIC}=\text{p}_{\text{D}}+\text{D}$, where the first term represents model complexity (the effective number of parameters) and the second term represents the goodness-of-fit. The fit of the model is better with smaller DIC values.

The models performed well; the trace plots showed good mixing of the three chains for each parameter (supplementary figures 1a and b.). Further, the chains reached a statistically stationary distribution and showed no evidence of auto-correlation. The Gelman-Rubin potential scale reduction parameter factor (PSRF) statistic, which provides a measure of MCMC convergence was less than 1.01 for all parameters. Gelman-Rubin PSRF values substantially greater than 1 indicate lack of convergence [^8^](#_ENREF_8).

**Text S2.** Required sample size determination.

The required sample size in a study is a function of the true prevalence, the diagnostic test performance and the required precision. To estimate the impact of the different herd outbreak prevalences in our dataset, we calculated the sample size for each herd outbreak that gives 80% power to estimate the Hui-Walter parameter estimates at the 0.05 level of significance (two sided) using the following formula:

$$n=\left(\frac{1.96}{d}\right)^{2}\left(\frac{\left[Se_{PI}\times p\right]+\left[1-Sp_{PI}\right]\left[1-p\right]}{Se_{PI}+Sp_{PI}-1}\right)\left(\frac{1-\left[\left[Se_{PI}\times p\right]+\left[1-Sp_{PI}\right]\left[1-p\right]\right]}{Se_{PI}+Sp_{PI}-1}\right)$$

where Se and Sp are the diagnostic test sensitivity and specificity following parallel interpretation, p is the true prevalence (p) and d is the absolute proportional error . The sample size per outbreak was calculated as the total number of cows minus the number of cows not inspected at the abattoir. The sensitivity (Se_PI_) and specificity (Sp_PI_) following parallel interpretation [^4^](#_ENREF_4) of the SICTT and abattoir inspection using estimates from the literature (Se_SICTT_=0.85, Sp_SICTT_=0.995 [^1^](#_ENREF_1); Se_Abattoir_=0.47 [^2^](#_ENREF_2), and Sp_Abattoir_=0.999 [assumed]) and outbreak-specific true prevalence [^11^](#_ENREF_11) were calculated and implemented in the sample size formula. All outbreaks with sample sizes less than the outbreak-specific required sample size were deleted.

**Text S3.** Bayesian jackknife and bootstrap analyses.

In the jackknife procedure, in each round one of the *n* outbreaks was omitted from the study dataset, and point estimates were computed using data from the remaining *n*-1 outbreaks. This process was repeated until jackknife-parameters were estimated for all 41 omissions from the original data set. Sampling bias (the systemic distortion of the parameter estimates from the true values) *b*, in standard deviation [σ] units) in the jackknife estimates (*p_n_*) from the parameter estimates from the full the study dataset (*p_full_*) was calculated as follows:

$$b_{n}=\frac{(p_{full}-p_{n})}{\sigma_{full}}$$

In the bootstrap procedure, 75% of the herd outbreaks were drawn randomly from the study dataset, without replacement (the Hui-Walter latent class model assumes that prevalence varies across subpopulations), the model was refitted to each of the bootstrap datasets and the point estimates were computed. This was repeated 100 times to derive the empirical estimate from the sampling distribution. The 95% bootstrap confidence intervals for the parameter estimates were obtained from the lower 5^th^ and upper 95^th^ percentiles tails of the empirical bootstrap distribution. The jackknife and bootstrap procedures were implemented using WinBUGS and the R2Winbugs package.

**
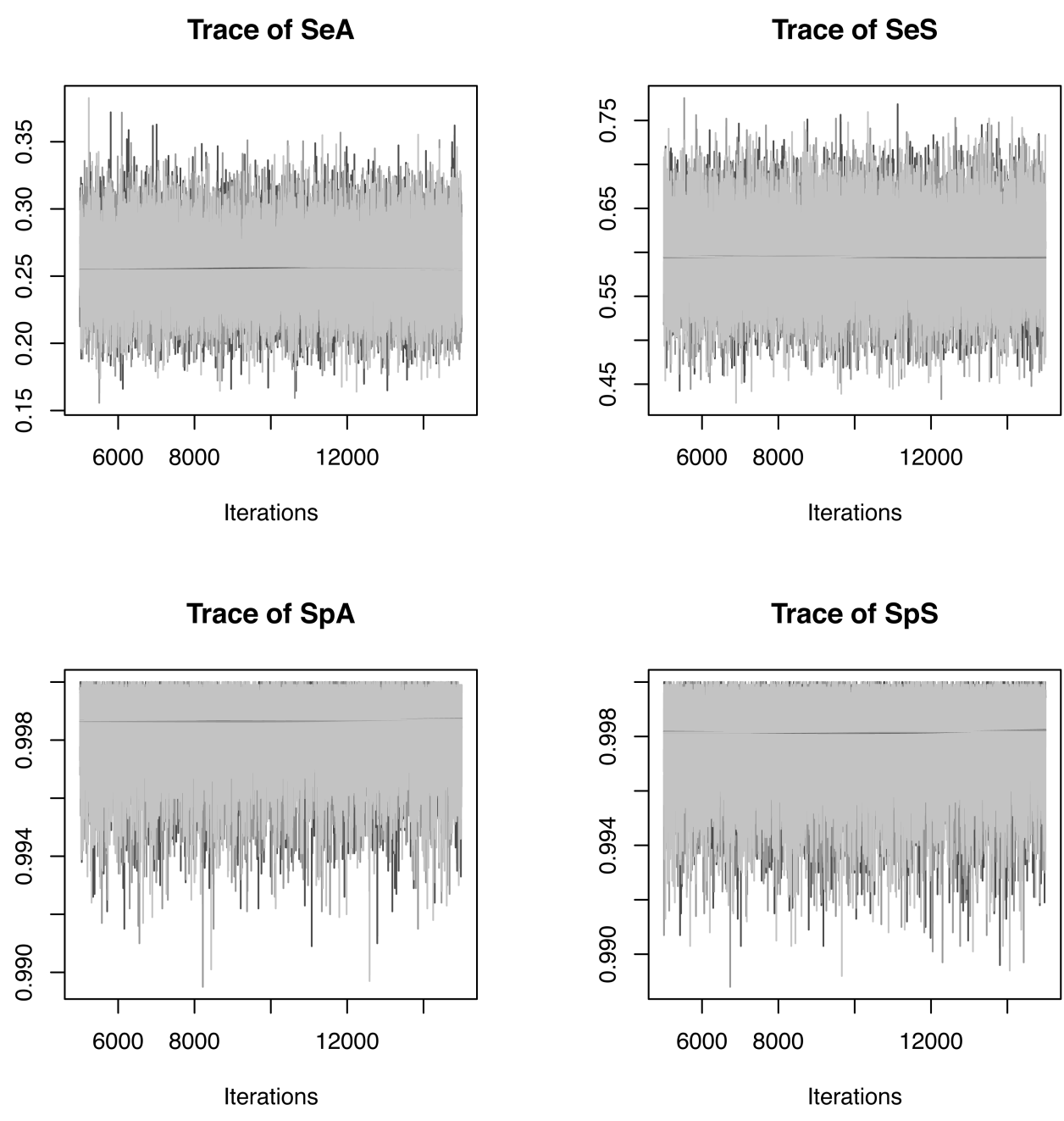
**

**Supplementary figure 1a. Trace plots of the diagnostic parameters of the single intradermal comparative tuberculin test (SICTT) and abattoir inspection.** The Markov chain Monte Carlo history plots for sensitivity (Se) and specificity (Sp) parameter estimates for the SICTT (S) and abattoir inspection (A) from the conditional independence model which included the outbreak specific diagnostic sensitivities. The plots record every 50^th^ sample from 500,000 iterations. The x-axis is the sequence of iterations and the y-axis the parameter values.


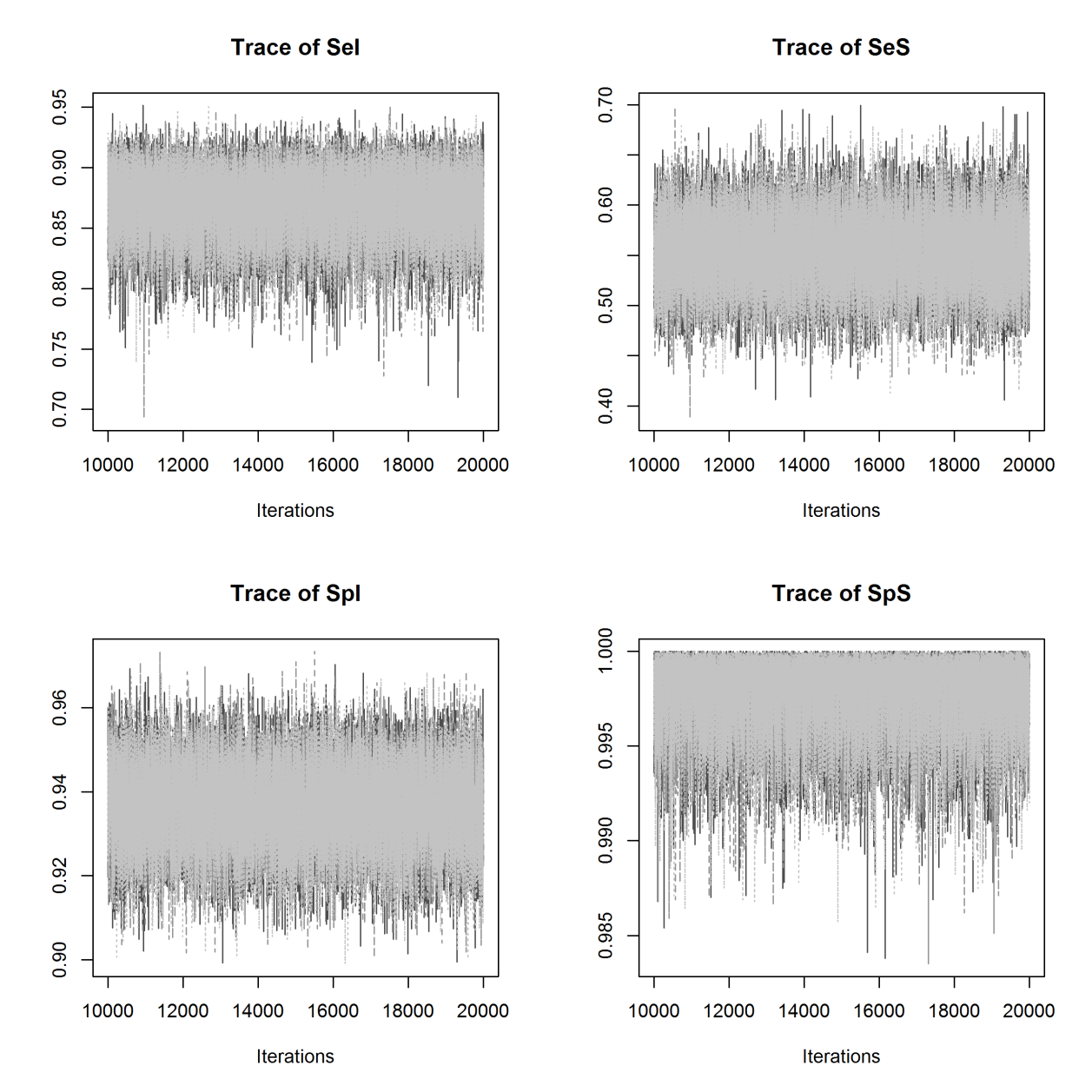


**Supplementary figure 1b. Trace plots of the diagnostic parameters of the single intradermal comparative tuberculin test (SICTT) and interferon(IFN)-γ assay.** The Markov chain Monte Carlo history plots for sensitivity (Se) and specificity (Sp) parameter estimates for the SICTT (S) and interferon(IFN)-γ assay (I) from the conditional independence model which included the outbreak specific diagnostic sensitivities. The plots record every 50^th^ sample from 500,000 iterations. The x-axis is the sequence of iterations and the y-axis the parameter values.

**Supplementary Results:**

**Table S1.** Parameter estimates of diagnostic accuracy (with 95% Bayesian credibility intervals [BCI]) for the of diagnostic sensitivity (Se) and specificity (Sp) for of the SICTT (1) and abattoir inspection (2), and true prevalence (P) with their empirical bootstrap distribution means and their 95% empirical bootstrap confidence intervals (EBCI) estimated from the from the subpopulation set analysis of the surveillance data.

|  | Point analysis | | Bootstrap analysis | |
| --- | --- | --- | --- | --- |
|  | Surveillance data | | | |
| Parameter | Mean | 95% BCI | Mean | 95% EBCI |
| Se_1_ | 0.595 | 0.508-0.687 | 0.590 | 0.502-0.637 |
| Sp_1_ | 0.998 | 0.994-1.000 | 0.997 | 0.996-0.998 |
| Se_2_ | 0.256 | 0.205-0.310 | 0.261 | 0.208-0.318 |
| Sp_2_ | 0.999 | 0.995-1.000 | 0.998 | 0.997-0.999 |
| P | 0.135 | 0.117-0.155 | 0.135 | 0.118-0.150 |


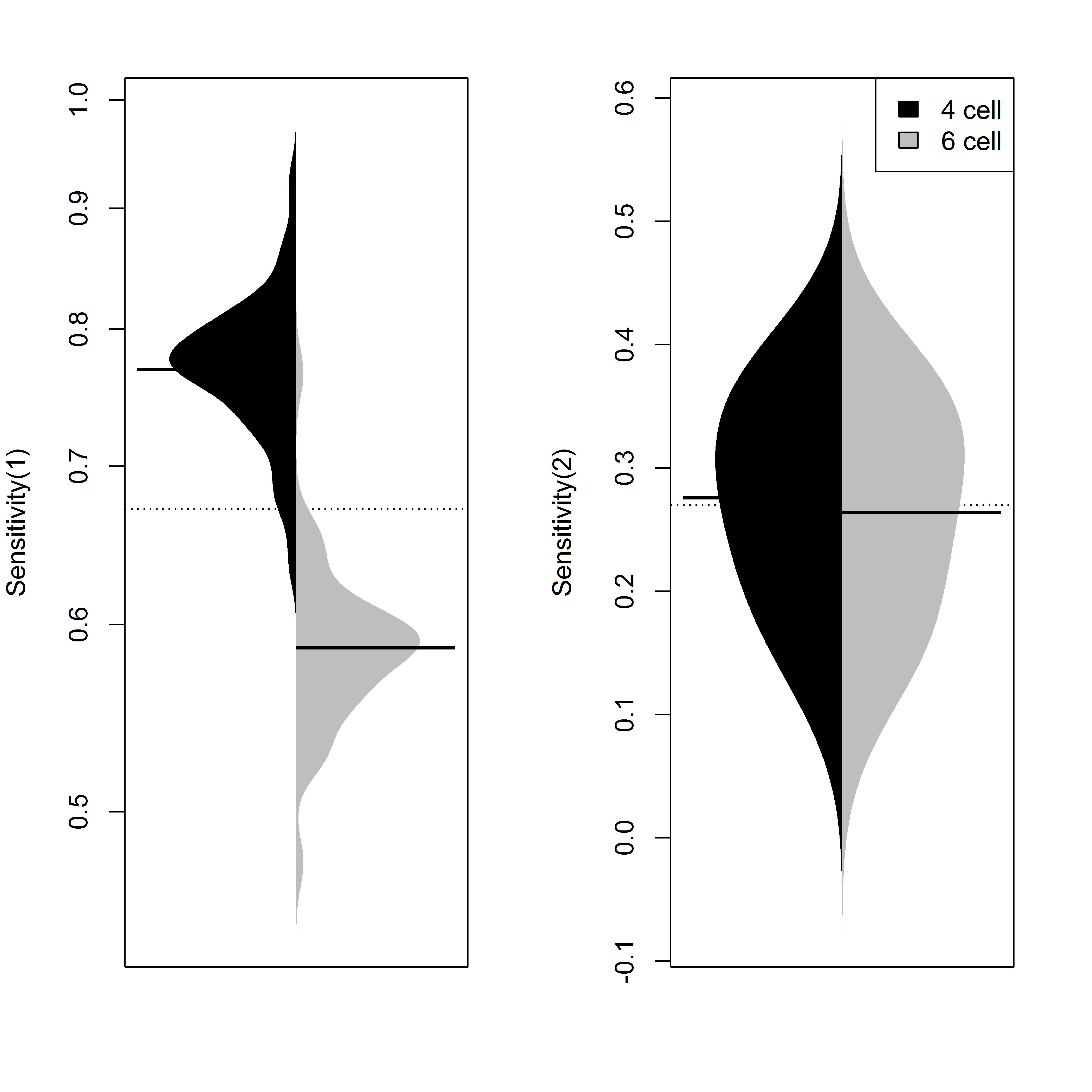


**Figure S1. Beanplots** **illustrating the variation in diagnostic test sensitivity across Northern Ireland** **tuberculosis herd outbreaks.** The resulting variation in the sensitivity of the single intradermal comparative tuberculin test (1) and abattoir inspection (2) from the traditional 4 and extended 6 cell conditional independence model including outbreak specific sensitivities across the 41 Northern Ireland tuberculosis herd outbreaks.


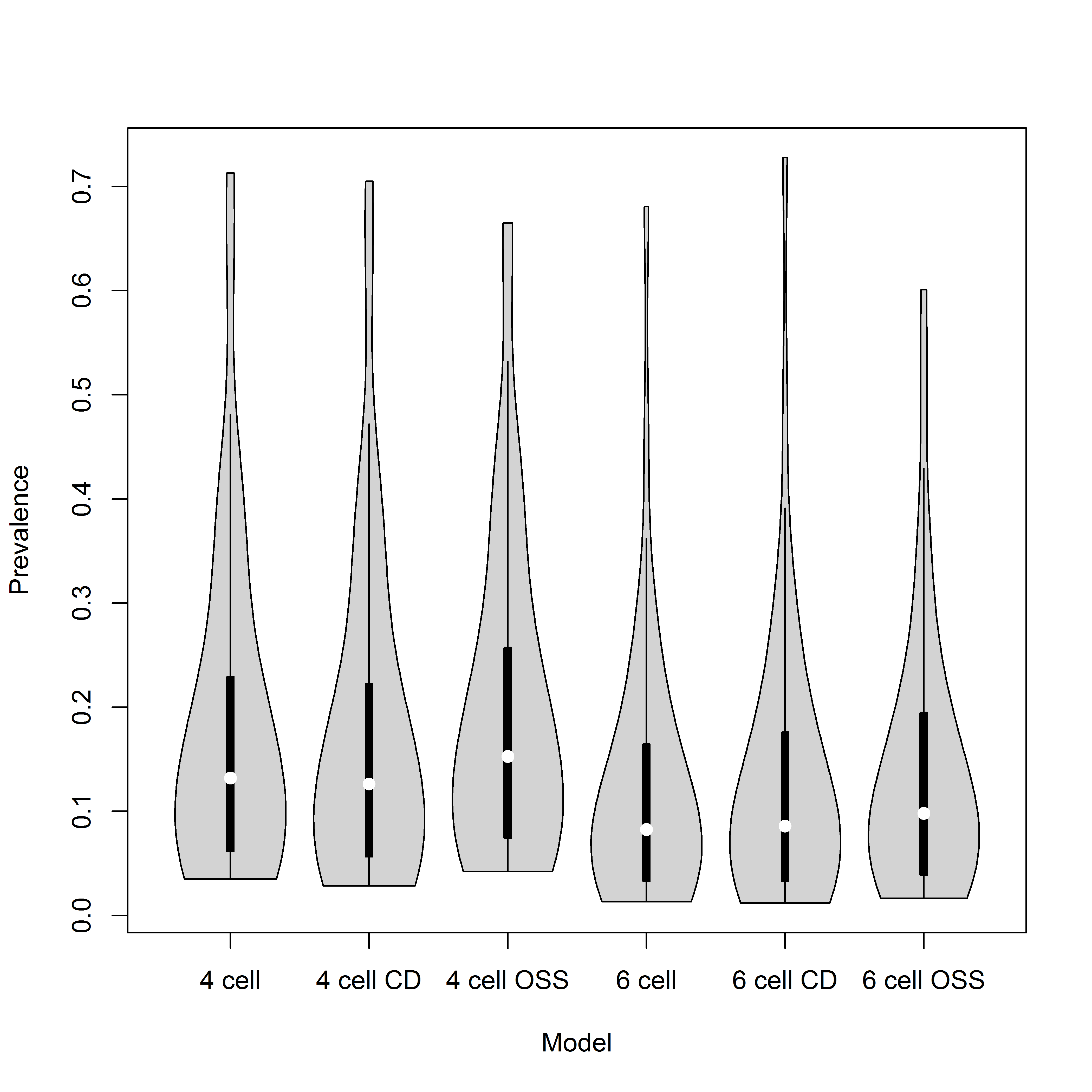


**Figure S2. Violin plots** **showing the variation in prevalence across** **Northern Ireland tuberculosis herd outbreaks.** The resulting variation in prevalence from the traditional 4 and extended 6 cell conditional independence and dependence (CD) models excluding/including outbreak specific sensitivities (OSS) across the 41Northern Ireland tuberculosis herd outbreaks.


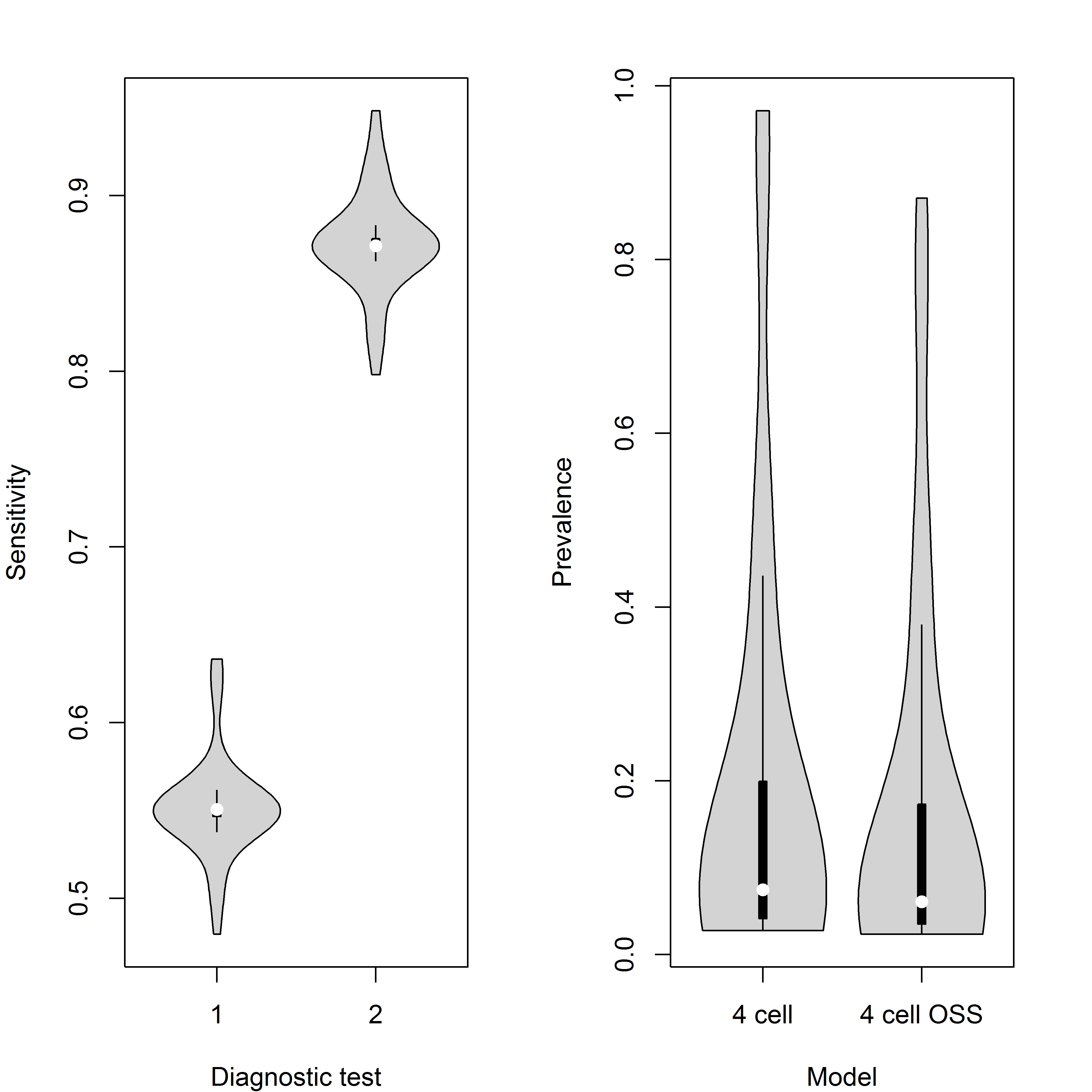


**Figure S3. Violin** **plots** **showing the variation in diagnostic test properties across Republic of Ireland** **tuberculosis herd outbreaks.** The resulting variation in the sensitivity of the single intradermal comparative tuberculin test (1) and interferon-γ assay (2) from the traditional 4 cell conditional dependence model including outbreak specific diagnostic sensitivities (OSS), and the prevalence from the traditional four cell conditional dependence model excluding/including OSS across the 38 Republic of Ireland tuberculosis herd outbreaks [^12^](#_ENREF_12).

**References**

1. De la Rua-Domenech, R. et al. Ante mortem diagnosis of tuberculosis in cattle: A review of the tuberculin tests,[gamma]-interferon assay and other ancillary diagnostic techniques. *Res. Vet. Sci.* **81**, 190-210 (2006).

2. Corner, L. et al. Efficiency of inspection procedures for the detection of tuberculous lesions in cattle. *Aust. Vet. J.* **67**, 389-392 (1990).

3. Dendukuri, N. & Joseph, L. Bayesian approaches to modeling the conditional dependence between multiple diagnostic tests. *Biometrics* **57**, 158-167 (2001).

4. Gardner, I., Stryhn, H., Lind, P. & Collins, M. Conditional dependence between tests affects the diagnosis and surveillance of animal diseases. *Prev. Vet. Med.* **45**, 107-122 (2000).

5. Lunn, D., Thomas, A., Best, N. & Spiegelhalter, D. WinBUGS-a Bayesian modelling framework: concepts, structure, and extensibility. *Stat Comput* **10**, 325-337 (2000).

6. Sturtz, S., Ligges, U. & Gelman, A. R2WinBUGS: a package for running WinBUGS from R. *J Stat Softw* **12**, 1-16 (2005).

7. Brooks, S. & Gelman, A. Alternative methods for monitoring convergence of iterative simulations. *J Comp Graph Stat.* **7**, 434-455 (1998).

8. Plummer, M., Best, N., Cowles, K. & Vines, K. Output analysis and diagnostics for MCMC. *R package version 0.13-4* (2009).

9. Bronsvoort, B. et al. No Gold Standard Estimation of the Sensitivity and Specificity of Two Molecular Diagnostic Protocols for Trypanosoma brucei spp. in Western Kenya. *PLoS ONE* **5**, e8628 (2010).

10. Humphry, R., Cameron, A. & Gunn, G. A practical approach to calculate sample size for herd prevalence surveys. *Prev. Vet. Med.* **65**, 173-188 (2004).

11. Bishop, S. & Woolliams, J. On the Genetic Interpretation of Disease Data. *PLoS ONE* **5**, e8940 (2010).

12. Clegg, T.A. et al. Using latent class analysis to estimate the test characteristics of the γ-interferon test, the single intradermal comparative tuberculin test and a multiplex immunoassay under Irish conditions. *Vet. Microbiol.* **151**, 68-76 (2011).
